# Oligomerization of Peptides LVEALYL and RGFFYT and Their Binding Affinity to Insulin

**DOI:** 10.1371/journal.pone.0065358

**Published:** 2013-06-21

**Authors:** Hsin-Lin Chiang, Son Tung Ngo, Chun-Jung Chen, Chin-Kun Hu, Mai Suan Li

**Affiliations:** 1 Department of Physics, National Tsing Hua University, Hsinchu, Taiwan; 2 Institute of Physics, Academia Sinica, Nankang, Taipei, Taiwan; 3 Institute for Computational Science and Technology, 6 Quarter, Linh Trung Ward, Thu Duc District, Ho Chi Minh City, Vietnam; 4 Institute of Physics, Polish Academy of Sciences, Warsaw, Poland; 5 Life Science Group, Scientific Research Division, National Synchrotron Radiation Research Center, Hsinchu, Taiwan; 6 Institute of Biotechnology, National Cheng Kung University, Tainan City, Taiwan; University of Akron, United States of America

## Abstract

Recently it has been proposed a model for fibrils of human insulin in which the fibril growth proceeds via stacking LVEALYL (fragment 11–17 from chain B of insulin) into pairs of tightly interdigitated 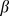-sheets. The experiments have also shown that LVEALYL has high propensity to self-assembly and binding to insulin. This necessitates study of oligomerization of LVEALYL and its binding affinity to full-length insulin. Using the all-atom simulations with Gromos96 43a1 force field and explicit water it is shown that LVEALYL can aggregate. Theoretical estimation of the binding free energy of LVEALYL to insulin by the molecular mechanic Poisson-Boltzmann surface area method reveals its strong binding affinity to chain B, implying that, in agreement with the experiments, LVEALYL can affect insulin aggregation via binding mechanism. We predict that, similar to LVEALYL, peptide RGFFYT (fragment B22-27) can self-assemble and bind to insulin modulating its fibril growth process. The binding affinity of RGFFYT is shown to be comparable with that of LVEALYL.

## Introduction

Study of protein aggregation is of paramount importance as it is associated with a number of diseases such as Alzheimer's disease, Hungtinton disease, spinocerebellar ataxia, type II diabetes [1]–[5] etc. Insulin is one type of a protein hormone that consists of chain A (21 residues) and chain B (30 residues) [6] connected by two inter-disulfide bonds. Its native state is a predominantly 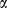-helical state. Type II diabetes is associated with insulin resistance and reduced insulin secretion [7]. Fibrils of full-length insulin are found at the site of frequent insulin injection [8] and possibly in patients with Parkinson's disease [9]. Insulin aggregation deteriorates its storage for long-term treatment of diabetes. Therefore, understanding of insulin fibrillation could offer safer storage and better treatment of Parkinson's disease.

Since theoretical study of fibrillation of full-length insulin is beyond present computational facility, several groups have considered its fragments such as LYQLEN (A13-18), LVEALY (B11-16) and VEALYL (B12-17). These peptides are highly prone to aggregation [10], [11]. Molecular dynamics (MD) simulations reveal that VEALYL peptides self-assemble into barrel-like oligomer [12]. Chains A and B are capable of forming fibrils on their own [13] and seeds of A or B can nucleate the fibril growth of full-length insulin [14].

Despite a lot of efforts the molecular structure of insulin fibrils has not been resolved yet. Insulin molecule is indicated to convert to 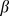-sheet and assemble to fibrillar structure from native state in vitro at low pH and elevated temperatures [15], [16]. A kinetic x-ray solution scattering study [17] suggested that insulin fibrils are formed by primarily 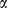-helical oligomers. Recently, Ivanova *et al.* [18] have reported a number of interesting experimental observations. First, aggregates of LVEALYL peptide (fragment B11-17) have fibrillar morphology. Second, based on this result they proposed a molecular model for full-length insulin fibrils, where the crystal structure of the fragment LVEALYL provides the basic structural organization of the spine of insulin fibrils. Third, LVEALYL is the key factor that can affect aggregation rates of insulin. While LVEALYL concentration is low, LVEALYL peptides nucleate to form the cross-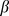 spine and serve as the template of amyloid fibril of insulin [19], [20]. However, it inhibits the fibrillation by preventing insulin molecules from attachment to the spine when the concentration is above the critical value [18], [21].

Motivated by experimental results of Ivanova *et al.* [18], in this paper we study self-assembly of peptide LVEALYL and its binding affinity to full-length insulin. Using all-atom simulations with the Gromos 43a1 force field and the simple point charge (SPC) water model, we show that LVEALYL forms antiparallel fibril. Since the direct probe of inhibition of insulin aggregation by this peptide is beyond present computational facilities, we have considered its binding affinity to insulin. With the help of the molecular mechanic Poisson-Boltzmann surface area (MM-PBSA) method [22] it is shown that LVEALYL displays high propensity to association with insulin. This result is qualitatively consistent with the experimental finding [18] that LVEALYL affects insulin fibrillation. Our study reveals the mechanism behind this effect that LVEALYL modulates aggregation rates by enhancing the beta-content (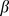-content) of insulin in monomer state.

Since LVEALYL is strongly bound to fragment RGFFYT from chain B (B22-27) we have also studied this fragment in detail. Our all-atom MD simulations predict that, similar to LVEALYL, RGFFYT self-assembles into fibril with antiparallel ordering. The binding free energy 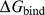 of this peptide to insulin has been also estimated by the MM-PBSA method. Since the binding affinity is relatively high RGFFYT can modulate insulin aggregation via the binding mechanism.

## Materials and Methods

### Crystal structures of insulin and peptide LVEALYL

The crystal structure of full-length human insulin used in our simulations was taken from Protein Data Bank (PDB) with PDB ID 1GUJ. This structure it was resolved at pH = 2.1 with 1.6 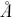 resolution [23]. The sequence of chain A contains 21 amino acids GLY ILE VAL GLU GLN CYS CYS THR SER ILE CYS SER LEU TYR GLN LEU GLU ASN TYR CYS ASN, while 30 amino acids of chain B are PHE VAL ASN GLN HIS LEU CYS GLY SER HIS LEU VAL GLU ALA LEU TYR LEU VAL CYS GLY GLU ARG GLY PHE PHE TYR THR PRO LYS THR. Peptide LVEALYL is the 11–17 segment of chain B that has PDB code 3HYD [18].

### Docking method

To dock LVEALYL and RGFFYT to insulin, we prepare PDBQT file for protein and peptide using AutodockTools 1.5.4 [24]. The Autodock Vina version 1.1 [25] which is much more efficient than Autodock 4 has been employed. In the Autodock Vina the Broyden-Fletcher-Goldfarb-Shanno method [26] is employed for local optimization. To obtain reliable results, the exhaustiveness of global search was set equal 400 while the maximum energy difference between the best binding mode and the worst one is chosen to be 7. The center of the grid was placed at the center of mass of insulin, with the grid dimensions 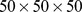 Å. These dimensions are large enough to cover the whole protein. Twenty binding modes (20 modes of docking) were generated with random starting positions of LVEALYL and RGFFYT which have fully flexible torsion degrees of freedom.

### MD simulations

The GROMACS 4.0.5 package [27] was used to run MD simulations with the GROMOS96 43a1 force field [28] and the SPC water model [29]. This force field was proved to be useful in studying folding [30] and aggregation [31]–[33] of peptides. The equations of motion were integrated by using a leap-frog algorithm [34] with a time step of 2 fs. The LINCS algorithm [35] was used to constrain the length of all bonds. The van der Waals (vdW) forces were calculated with a cut-off of 1.4 nm, and particle-mesh Ewald method [36] was employed to treat the long-range electrostatic interactions. The non-bonded interaction pair-list was updated every 10 fs with the cut-off of 1 nm. The V-rescale temperature coupling, which uses velocity rescaling with a stochastic term [37], has been used to couple each system to the heat bath with a relaxation time of 0.1 ps. The Berendsen pressure coupling method [38] was applied to describe the barostat with constant pressure at 1 atm. Production MD simulations have been performed in NPT ensemble at 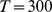 K and pH = 7.

The dynamics of LVEALYL and RGFFYT dimers has been studied to probe their oligomerization. For each of these systems we performed four 150–300 ns MD simulations. Initial conformations were generated by randomly placing peptides in periodic boxes. The concentration of LVEALYL and RGFFYT is about 52 mM that is about three orders of magnitude higher than those used in experiments.

To see if the crystal structure, obtained at pH = 2.1 [23], remains stable at pH = 7, we have carried 300 ns MD simulation for monomer insulin starting from its PDB structure [23]. The 300 ns MD runs has been also performed for the complex of insulin and fragment LVEALYL. This simulation is aimed at probing the effect of LVEALYL on secondary structures and stability of full-length insulin.

### MM-PBSA method

The details of MM-PBSA method are given in Information S1. Overall, in this method the binding free energy of ligand to receptor is defined as follows
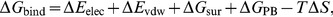
(1)where 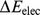 and 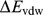 are contributions from electrostatic and vdW interactions, respectively. 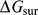 and 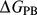 are nonpolar and polar solvation energies. The entropic contribution 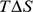 is estimated using the normal mode approximation (Information S1). In order to calculate 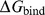 the MD simulations have been carried out using the Gromos force field 43a1 and the SPC model of water.

For estimation of 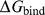 of LVEALYL and RGFFYT to insulin four 100–150 ns MD simulations were performed starting from the configuration obtained in the best docking mode but with different random iseed numbers. Snapshots collected in equilibrium have been used for estimating 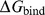 by the MM-PBSA method (Eq. 1).

### Tools and measures used in the structure analysis

#### Order parameter 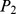


The nematic order parameter 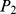 [31], [39] is used to characterize the fibril state of short peptides. If 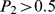, then a system has the propensity to be in an ordered state. In this paper we calculate 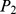 for dimer of LVEALYL and RGFFYT. The fibril state of these peptides is formed if one observes two antiparallel 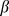-strands by using Visual Molecular Dynamics(VMD) [40] and this happens at 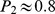.

#### Free energy landscape

The free-energy surface along the 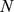-dimensional reaction coordinate 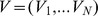 is given by 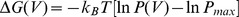, where 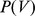 is the probability distribution obtained from a histogram of the molecular dynamics (MD) data, 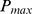 is the maximum of the distribution, which is subtracted We will compute the free energy landscapes (FEL) of LVEALYL dimer as a function of 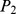 and cosine of the angle between two end-to-end vectors of peptides.

#### Contact maps

The time evolution of formation of side chain-side chain (SC-SC) and hydrogen bond (HB) contacts was monitored. A SC-SC contact is formed if the distance between the centers of mass of two residues is 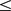
6.5 Å. HB is formed if the distance between donor D and acceptor A is 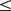
3.5 Å and the angle D-H-A is 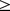 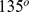. The number of HBs is computed using the Gromacs software.

#### Secondary structures

Secondary structure contents have been estimated by STRIDE [41] algorithm. Plots for molecular structures are made by VMD [40] and the Python-enhanced molecular graphics tool (PyMOL) [42].

## Results and Discussion

### Oligomerization of LVEALYL

#### Antiparallel ordering of dimer 2LVEALYL

**Figure 1:**
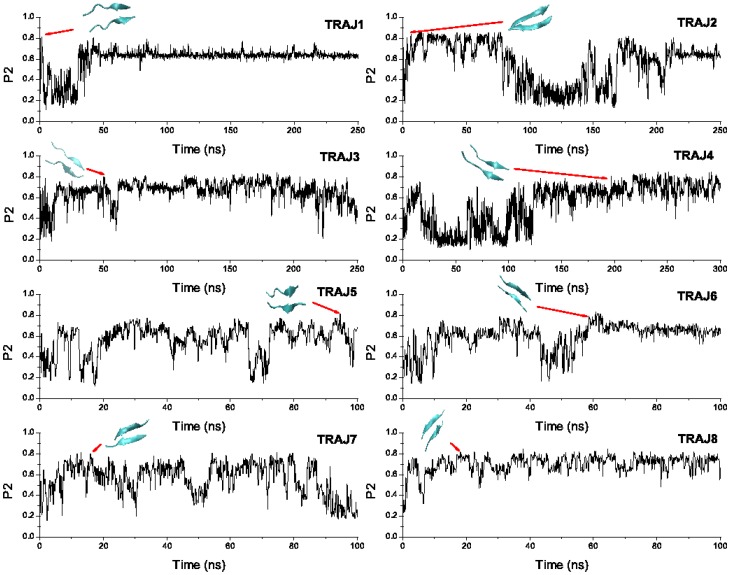
Time dependence of order parameter 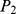 for LVEALYL dimer. The order parameter 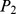 has been calculated using the definition of Nguyen et al [31]. Fibril structures are formed at 1.6, 6.0, 50.4, 196.7, 94.6, 60.6, 15.9, and 18.1 ns for run 1, 2, 3, 4, 5, 6, 7, and 8, respectively.

**Figure 2:**
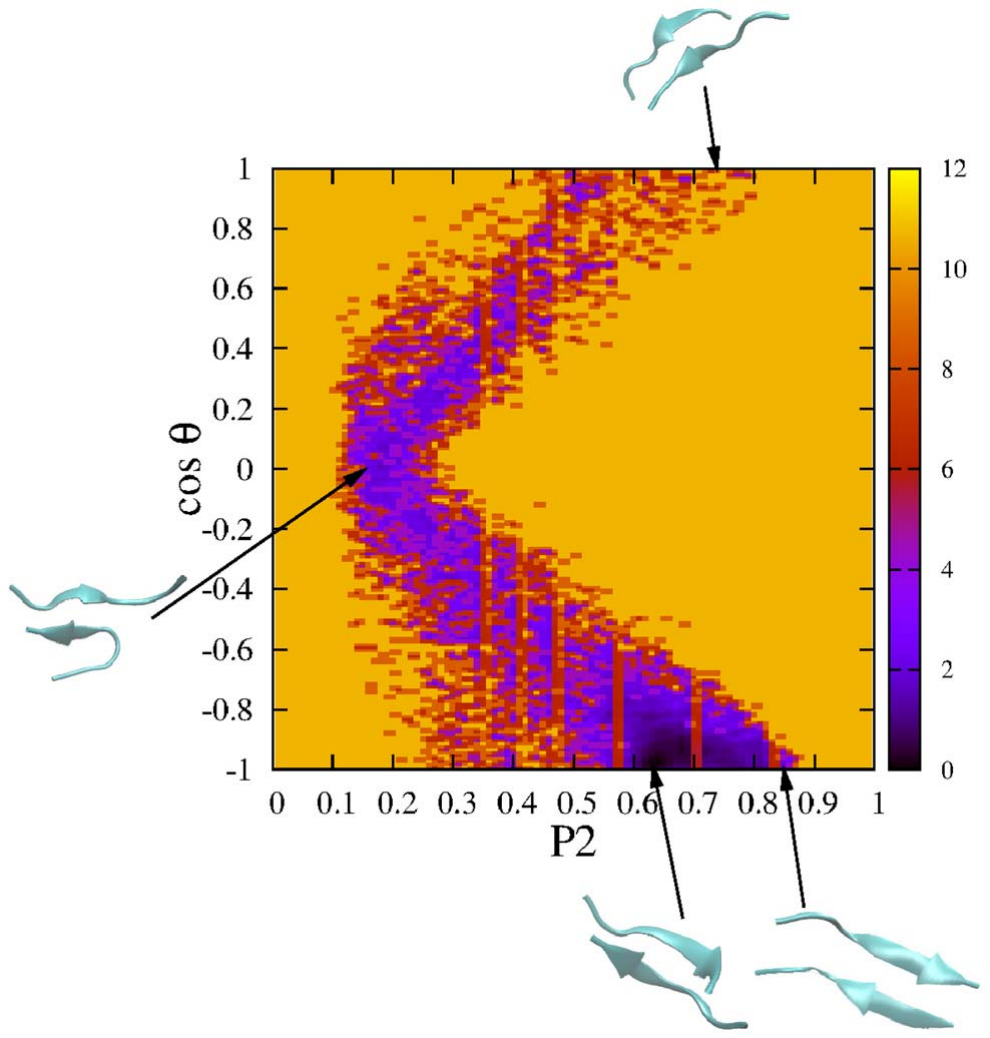
Two-dimensional FEL of LVEALYL dimer. 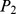
 and 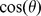 are used as reaction coordinates for construction of FEL. The results were obtained using snapshots collected in eight MD trajectories shown in Figure 1. Typical snapshots are also shown.

To probe the propensity of LVEALYL to fibrillation we have carried 8 MD runs starting from random conformations. The time dependence of the order parameter 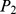 is shown in Figure 1. The diversity of oligomerization pathways is clearly seen as the system reaches the ordered phase at different time scales. In the first and second runs the fibril state appears almost immediately without intermediates. The antiparallel conformation also occurs quite rapidly in trajectory 7 and 8. For third, fourth, fifth and sixth trajectories several intermediate states that correspond to long-lived plateaus occur on routes to the ordered state. In order to show that the fibril with antiparallel ordering is more favorable than parallel arrangement, we plot FEL as a function of 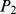 and 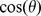, where 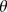 is the angle between two end-to-end vectors of peptides. The occurrence of the dominant local minimum at 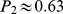 and 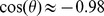 (Fig. 2) ascertains the dominance of antiparallel ordering. The parallel conformations appear during simulation but with very low probability (see also Figure S1). In the experiment of Ivanova *et al* [18], LVEALYL peptides are parallel within one beta sheet but peptides from different beta sheets are antiparallel. The difference between our result and the experiment is caused by different pH. In our simulations pH = 7 and at this pH among 7 amino acids only third residue Glutamic acid (E) bears negative charge. This leads to antiparallel ordering which minimizes electrostatic repulsion between Glutamic acids from different chains. At pH = 2.1 that used in the experiment Glutamic acid becomes neutral and the parallel arrangement in one beta sheets is more favorable. This is also confirmed by our preliminary results on low pH simulations (ST Ngo et al, unpublished results).

Assuming that the fibril of dimer is formed if 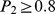 we obtain the fibril formation time 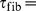
1.6, 6.0, 50.4, 196.7, 94.6, 60.6, 15.9, and 18.1 ns for run 1, 2, 3, 4, 5, 6, 7, and 8, respectively (Figure 1). To ascertain that in the fibril state two chains form the beta sheet we calculate the number of backbone HBs. One can show that for 8 snapshots shown in Figure 1 the number of inter-chain backbone-backbone HBs varies between 3 and 7 (see one example in Figure S2). According to the standard definition (http://en.wikipedia.org/wiki/Beta_sheet), the beta sheet is formed if the number of backbone-backbone HBs is larger or equal 2. Thus, in the fibril state with 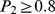 the dimer forms the beta sheet. Averaging over 8 runs one has 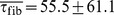 ns. Having used the same force field and water model we have previously obtained 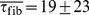 ns for dimer of the fragment A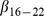 from amyloid beta peptides [43]. Thus the dimerization of A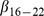 is faster than LVEALYL due to difference in sequences. The rapid fibrillation of A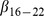 is associated with two opposite charges at the ends [44], [45].

It is well know that the 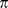
−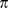 interactions may enhance the fibrillogenesis of polypeptide chains [46]. From this prospect the high propensity to aggregation of LVEALYL is presumably associated with the aromatic ring of Tyrosine (Y). To clarify this point we have calculated the interaction energies between pairs of residues from two chains as a sum of the vdW and electrostatic energies (Figure S3). Clearly, the L(Leu)-L(Leu) interaction dominates but not the Y-Y interaction which is even weaker than the Y-L interaction. This is because the distance between two Tyr residues is large due to the antiparallel ordering. The fact that the 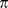
−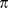 interaction does not play the critical role is in line with the recent study [47] showing that the mutation of Phenylalanine at position 19 and 20 by Leu or Ile even promotes aggregation of the Alzheimer's A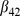 peptide. It would be interesting to see how the mutation of Tyrosine by some residue which lacks the aromatic ring changes the kinetics of LVEALYL aggregation.

#### Side chain interaction is more important than hydrogen bond interaction

To probe the nature of ordering of 2LVEALYL, we have constructed SC and and HB contacts maps (Figure S4) using snapshots collected in eight MD runs (Fig. 1). These contacts maps clearly show that two peptides are aligned in antiparallel manner having contacts between residues from different terminals more populated than those from the same terminal. As expected, the SC contact between middle Alanines (A) is the most probable (Figure S4). Since the probability of observing HB contacts is lower than that of SC contacts one can conclude that the SC interaction (Figure S4) dominates over HB interaction. The similar behavior has been also observed for other short peptides [32], [43], [44].

### Binding of LVEALYL to insulin

#### Structure of monomer insulin at pH = 7

**Figure 3:**
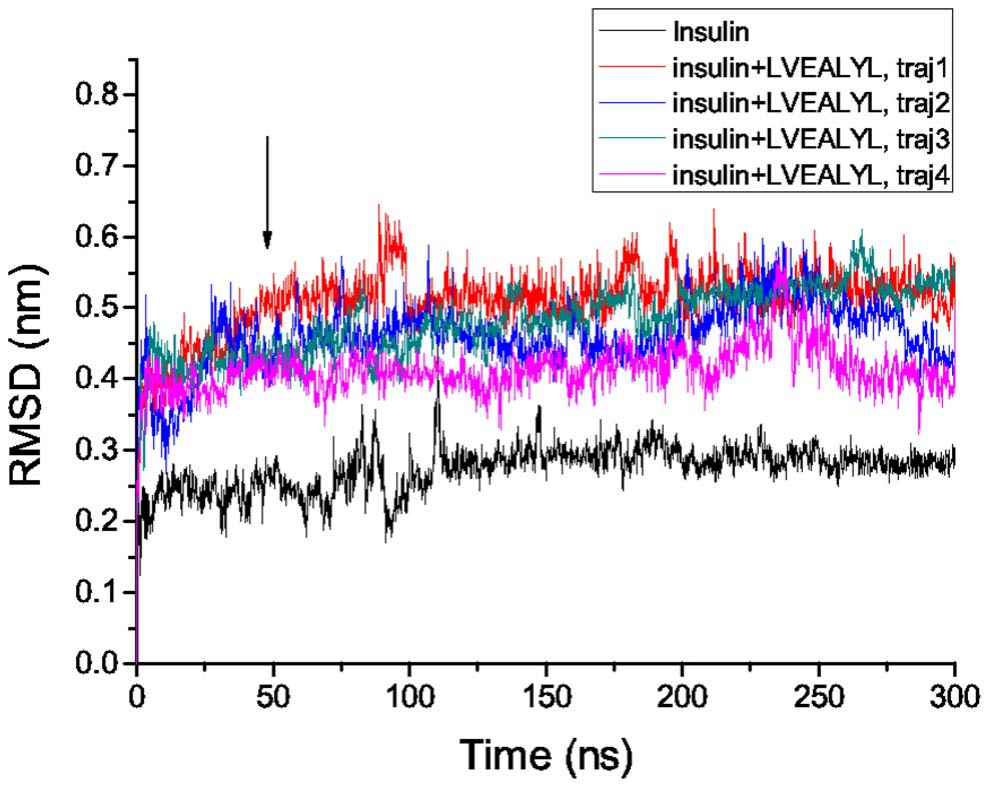
Time dependence of root mean square deviation of insulin. The C-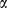 rmsd of monomer insulin (black) and insulin in the complex with LVEALYL (color curves) was obtained during 300 ns MD simulations at pH 7. The structure resolved at pH 2.1 remains stable at pH 7. Arrow roughly refers to equilibration time 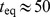 ns, when the system reaches equilibrium (curve saturation).

Because the structure of insulin at neutral pH is not available, in order to obtain a reasonable structure for docking and MM-PBSA simulations at this pH we have done the following. Using the PDB structure (PDB ID: 1GUJ) obtained at pH = 2.1 as a initial conformation 300 ns MD simulation has been carried out at pH = 7 and 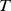 = 300 K. The time dependence of C*_α_*-root mean square displacement (rmsd) compared to the PDB structure is shown by the black curve in Fig. 3. Since the saturation value of rmsd is less than 0.3 nm, the NMR structure, obtained at pH = 2.1 [23], may be considered as stable at pH = 7. One of possible reasons behind this is that insulin has two inter-disulfide bridges that keep chains A and B together and the intra-disulfide bridge between residues A6 and A11 enhances the stability of chain A. The high stability of monomer is consistent with Pease's suggestion [48] that insulin monomer never transform it's helix structure to 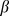-rich structure and variations of second structures are processed when insulin is attached to the end of fibril.

We use the C*_α_* rmsd conformational clustering method [49] implemented in the Gromacs software to screen out *in silico* dominant structures obtained in equilibrium. With the clustering tolerance of 0.18 nm we have obtained 3 clusters and the typical structure of the most populated cluster (99%) is shown in Figure S5. One can show that rmsd between this structure and PDB structure obtained at pH = 2.1 (PDB ID: 1GUJ) is 0.28 nm. The rmsd between PDB structure obtained at pH = 8.5 [50] (PDB ID: 3I3Z) and the most populated structure is 0.26 nm (Figure S6). Moreover, rmsd between 1GUJ and 3I3Z is about 0.32 nm implying the weak dependence of insulin structure on pH. Thus we will use PDB structure 1GUJ for further docking and MD simulations at pH = 7.0.

#### Docking result

**Figure 4:**
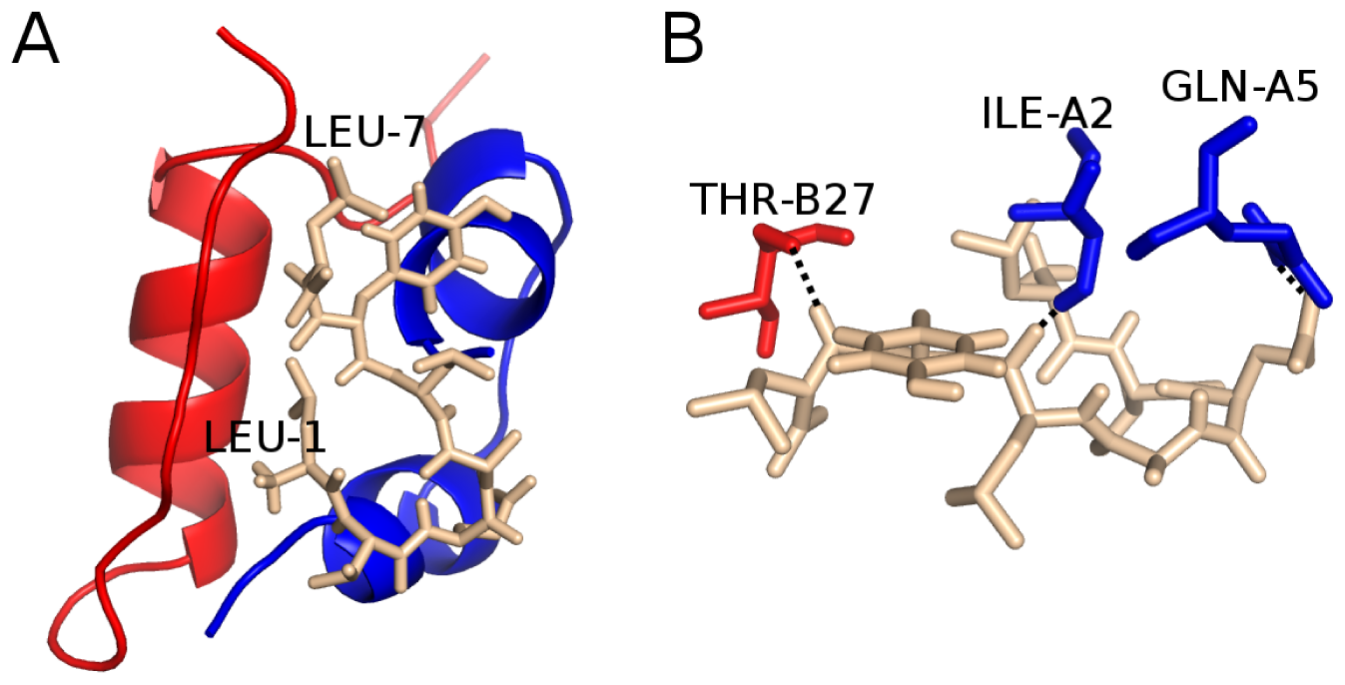
The best docking mode and hydrogen network between LVEALYL and insulin. (A) The lowest binding energy conformation of LVEALYL (brown) to insulin obtained by the docking method. Red and blue color refer to chain A and B, respectively. LEU-1 and LEU-7 are the first and last residues of LVEALYL. In the best docking mode the binding energy is 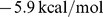. (B) In the best position, LVEALYL peptide is bound by three hydrogen bonds with ILE-A2, GLN-A5,and THR-B27 of the receptor, respectively.

Using the Autodock Vina we dock LVEALYL to the PDB structure 1GUJ. In the best docking mode (Fig. 4) the binding energy of LVEALYL to receptor is lowest and equal 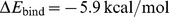. In this position, LVEALYL peptide forms three hydrogen bonds with residues ILE-A2, GLN-A5, and THR-B27 of insulin molecule (Fig. 4). Thus LVEALYL is bound to both chains A and B.

#### MM-PBSA result

Because the docking method is not accurate due approximations such as omission of receptor dynamics and a limited number of possible conformations of ligand, we apply the more precise MM-PBSA method to estimate the binding free energy 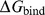. Four 300 ns MD simulation trajectories have been carried out for insulin-LVEALYL complex using the conformation generated by Autodock Vina in the best mode (Fig. 4A) as the initial configuration.

As evident from the time dependence of rmsd of insulin from its initial conformation (Fig. 3), the system reaches equilibrium (the curve gets saturation) at 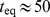 ns for all MD runs. Using snapshots collected in equilibrium (time window 50–300 ns) and MM-PBSA method we have estimated 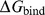 for four trajectories (Table 1). Within error bars we have also obtained the same result for time window 50–100 ns, showing that our MD runs are sufficient for estimation of the binding free energy. The electrostatic interaction is more important than the van der Waals interaction (Table 1). This is probably because both receptor and ligand have non-compensated charges.

**Table 1:** Binding free energies of LVEALYL and RGFFYT.

ligand	Traj	Δ*E_elec_*	Δ*E_vdw_*	Δ*G_sur_*	Δ*G_PB_*	*T*Δ*S*	Δ*G_bind_*
LVEALYL	1	−74.7	−48.4	−7.2	75.2	−41.3	−13.7
	2	−81.7	−56.2	−7.6	94.8	−39.8	−10.9
	3	−68.9	−67.2	−8.4	79.9	−40.3	−24.3
	4	−86.9	−46.8	−6.3	93.4	−38.4	−8.2
	Average	−78.1±6.8	−54.7±8.1	−7.4±0.8	85.8±8.5	−40.0±1.1	−14.3±6.1
RGFFYT	1	−172.7	−50.0	−6.5	187.4	−34.7	−7.2
	2	−143.3	−40.5	−5.2	143.9	−34.5	−10.5
	3	−95.1	−55.6	−6.3	108.3	−34.8	−13.9
	4	−195.0	−46.9	−5.5	188.6	−35.8	−22.9
	Average	−151.5±37.4	−48.3±5.5	−5.9±0.5	157.1±33.4	−35.0±0.5	−13.6±5.9

Binding free energy 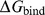 of two peptides to insulin obtained by the MM-PBSA method using Eq. 1 and snapshots collected in equilibrium of four MD trajectories. All values are given in 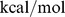.

We roughly estimate the inhibition constant IC_50_ which expresses the concentration of inhibitor required to produce 50 per cent inhibition of an enzymic reaction inhibition as follows. Assuming that the inhibitor is noncompetitive or the Michaelis constant 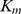 is much lower than the substrate concentration one has IC_50_ = *K_I_* [51], where the dissociation constant of the enzyme-inhibitor complex 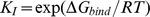 (note that 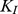 is also called the inhibition constant). Here gas constant 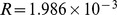 kcalmol^−1^K^−1^ and 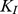 is measured in mol. Using 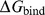 (Table 1) we obtain IC_5_0∼nM implying that LVEALYL strongly binds to insulin. This is in line with the experiments [18] that LVEALYL affects insulin aggregation substantially.

### Effect of LVEALYL on the secondary structure of insulin

To explore the effect of LVEALYL on the structural stability of insulin we study snapshots collected from four 300 ns MD runs (Fig. 3). In the presence of this short peptide rmsd of insulin from its initial structure becomes larger. In equilibrium average rmsd is about 0.48 nm implying that LVEALYL destabilizes the structure of full-length insulin.

#### LVEALYL increases 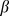-content of insulin

Using the STRIDE analysis, one can study the effect of LVEALYL on secondary structures of insulin. The time dependence of secondary structures obtained from four MD runs is shown in Figures S7, S8, S9, S10. We first check if our configuration sampling is sufficient for studying structure dynamics. For this aim one has to calculate the average secondary structure content in equilibrium using snapshots collected after 50 ns (Fig. 3) but for time windows 50–150 ns and 50–300 ns. As follows from Figure S11, the average 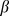-contents of insulin obtained from four MD trajectories are essentially the same for two time windows (Figure S11) (the same is valid for helix and coil but results not shown). This robust result indicates that snapshots generated in our simulation can capture dynamics of the insulin-LVEALYL complex.

**Figure 5:**
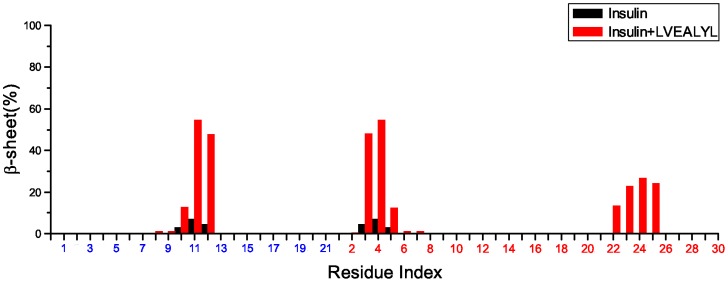
Average beta-content of insulin. Beta-content of each residue of insulin in the absence (black) and presence (red) of LVEALYL. Red and blue indices refer to chain A and B, respectively. The results are averaged over snapshots collected in equilibrium during one and four 300 ns MD simulations for insulin and insulin-LVEALYL complex, respectively.

**Figure 6:**
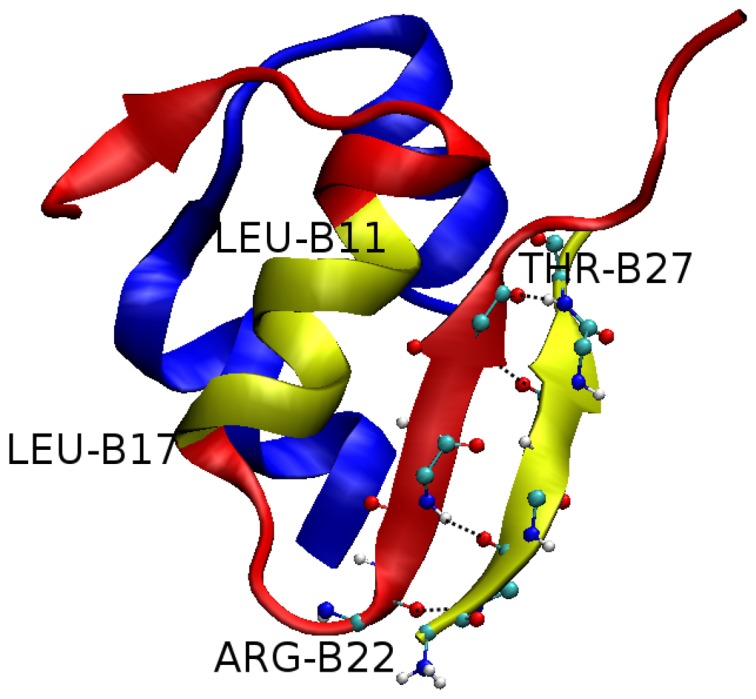
Dominant structure of insullin-LVEALYL complex. Structure of the most populated cluster (96%) from 12 clusters obtained by the clustering technique with tolerance of 0.2 nm. The result was obtained using snapshots collected in equilibrium during 300 ns MD simulations. LVEALYL peptide and fragment B11-17 are highlighted in yellow. Fragment B22-27 becomes a 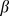-strand in the presence of LVEALYL, but not B11-17. LVEALYL forms the beta sheet with insulin having 4 backbone-backbone HBs.

Using results shown in Figures S7, S8, S9, S10, one can show that in the presence of LVEALYL the average helix content of insulin slightly decreases from 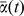 = 44.41% to 43.27%. However, the clear increase in 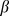-content is seen (Fig. 5) as it levels from 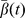 = 0.67% to 6.53%. It is high at residues 11 and 12 from chain A, and 3, 4 and 22–25 of chain B. Using the C*_α_*-rmsd conformational clustering technique implemented in the Gromacs software and snapshots collected in equilibrium of four 300 ns MD runs one can obtain representative structures of insulin. With the clustering tolerance of 0.2 nm we have obtained 12 clusters. Among them the most populated structure (96%) has two beta strands at B1-5 and B22-26 (Fig. 6). LVEALYL forms the beta sheet with fragment B22-27 having 4 backbone-backbone HBs.

It is known that the higher aggregation rates are the higher is the population of the fibril-prone conformation **N** ^*^ in monomer state [52]–[54]. One of the examples supporting this hypothesis is that A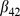 peptides aggregate into 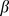-sheet fibril much faster than A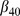 ones [55] because the former has higher 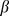-content in the monomer state [53], [56]. In the insulin case, the structure of **N** ^*^ conformation remains unknown but one may hypothesize that it has richer beta structure compared to the native state. Based on this hypothesis and on the increase of 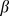-content of monomer insulin in the presence of LVEALYL one may expect that this peptide enhances the population of of **N** ^*^ conformation in monomer state promoting insulin aggregation. The experiments of Ivanova et al [18] has shown that the effect of LVEALYL on fibril elongation of insulin depends on the insulin∶LVEALYL ratio. The substantial inhibition is observed for insulin∶LVEALYL ratio equal 1∶1/10 and 1∶1. Since our simulation is carried out of the ratio 1∶1 one expects that LVEALYL would strongly prevent the fibril growth. From this prospect, our results contradict the experimental finding [18]. However, closer inspection of data presented in Fig. 5 shows that it is not necessary that our results are inconsistent with the experiment. This may be understood considering the following argument. The 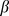-content of the most fibril-prone fragment B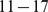 [18] is not promoted by LVEALYL (Fig. 5). The increase in 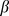-structure happens at residues 11 and 12 from chain A and 3, 4, and 22–25 from chain B. Since it remains unclear if these residues belong to fibril-prone regions one cannot be ascertained that this effect leads to promotion or inhibition of aggregation from the theoretical point of view. The correct answer would be provided by direct simulations of fibril formation of full-length insulin but this problem is beyond present computational facilities. Nevertheless, our results obtained for binding affinity and changes of secondary structures of insulin by LVEALYL suggest that this peptide modulate fibrillation rates. Finally, LVEALYL also slightly decreases the coil content from 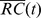 = 32.36% to 29.19% and the increase in 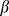-content of insulin occurs at expense of the decrease of the coil content (Figures S7, S8, S9, S10).

#### Fragment B11-17 does not adopt beta-strand in the presence of LVEALYL

One of the most interesting propositions made by Ivanova et al. is that fragment B11-17 (LVEALYL) can nucleate insulin aggregation in such a way that upon addition of a new insulin molecule B11-17 fragments of two insulins transform from helix to beta strands. Then two insulin molecules are attached to each other at this fragment initiating fibril growth. An interesting question emerges is that whether B11-17 fragment of monomer insulin adopts beta-strand in the presence of peptide LVEALYL. As evident from Fig. 5 and Fig. 6, there is no beta-strand extended over residues B11-17. The helix content remains essentially the same as in the absence of LVEALYL (Figure S12), but LVEALYL itself becomes the 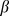-strand in the presence of monomer insulin (Fig. 6 and Movie S1).

Thus, the addition of LVEALYL does not convert helix fragment B11-17 into the beta one. However, this does not mean that the hypothesis of Ivanova *et al* [18] about the role of B11-17 segment as a template for fibril growth is not valid. To shed light on this problem one has to study the system which consists of at least two full-length insulin molecules. This problem requires further investigation.

#### LVEALYL is strongly bound to segment B22-27

**Figure 7:**
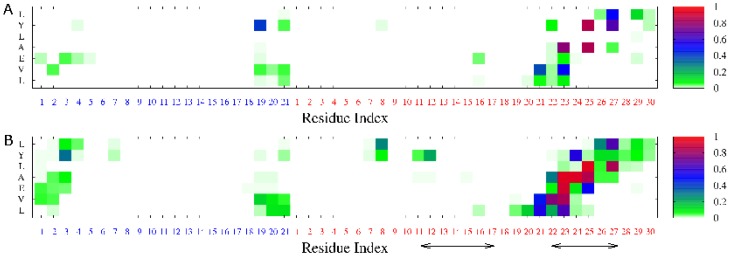
Contact maps for insulin-LVEALYL complex. Hydrogen bond (A) and side chain (B) contact maps of LVEALYL peptdie and insulin. Results were obtained in 300 ns MD simulations. Arrows refer to fragment B11-17 and B22-27.

As follows from Fig. 6 and Movie S1, during MD simulation LVEALYL spends most of time at the end of chain B. However, in order to know its location more precisely we construct the HB and SC contact maps between insulin and LVEALYL (Fig. 7). In difference from the dimer 2LVEALYL case where the side chain interaction dominates over the hydrogen bonding, there is no pronounced difference between contributions of these two interactions to binding affinity of LVEALYL to insulin. LVEALYL strongly interacts with residues B22-27 (Fig. 7). During 300 ns MD runs these residues appear to be in the extended beta conformation as evident from Figure S13 where the time dependence of the length of beta structure of fragment B22-27 is shown. Here the length of beta structure is defined as the number of residues in the beta conformation. We have also studied the survival probability of HBs that were formed in the best docking mode (Fig. 4) during MD course. In the best docking mode one has 3 HBs but this number fluctuates between 0 and 1 (Figure S14) as simulations progress. The difference between docking and MD results is associated with the fact that the docking adopts a number of major approximations such as omission of receptor dynamics.

### Binding of RGFFYT to insulin

Since upon binding to insulin LVEALYL is bound to fragment B22-27 (RGFFYT), one wonders about binding affinity of RGFFYT to insulin. The answer to this question would be useful for searching a new peptide to interfere with insulin aggregation.

#### Docking result

**Figure 8:**
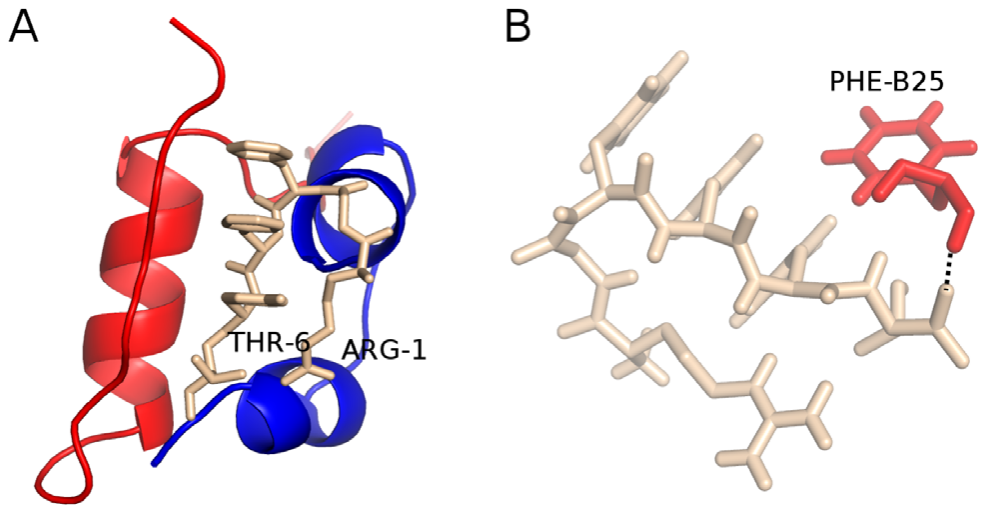
The best docking mode and hydrogen network between RGFFYT and insulin. (A) The best docking mode of peptide RGFFYT (brown) to insulin. ARG-1 and THR-6 are the first and last residues of RGFFYT. The binding energy 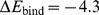 kcal/mol. (B) In this position, RGFFYT has one hydrogen bond with PHE-B25 of chain B.

Using the docking method we calculate the binding energy of RGFFYT to insulin. Fig. 8 shows the conformation obtained in the best mode with the binding energy 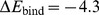 kcal/mol. This value is higher than that for LVEALYL, presumably because RGFFYT is shorter. In the best mode position RGFFYT is bound to insulin by one hydrogen bond with Phe-B25 with one piece parallel to the terminal part of chain B (Fig. 8). Interestingly, this peptide does not bind to fragment B11-17 with sequence LVEALYL.

#### MM-PBSA result

**Figure 9:**
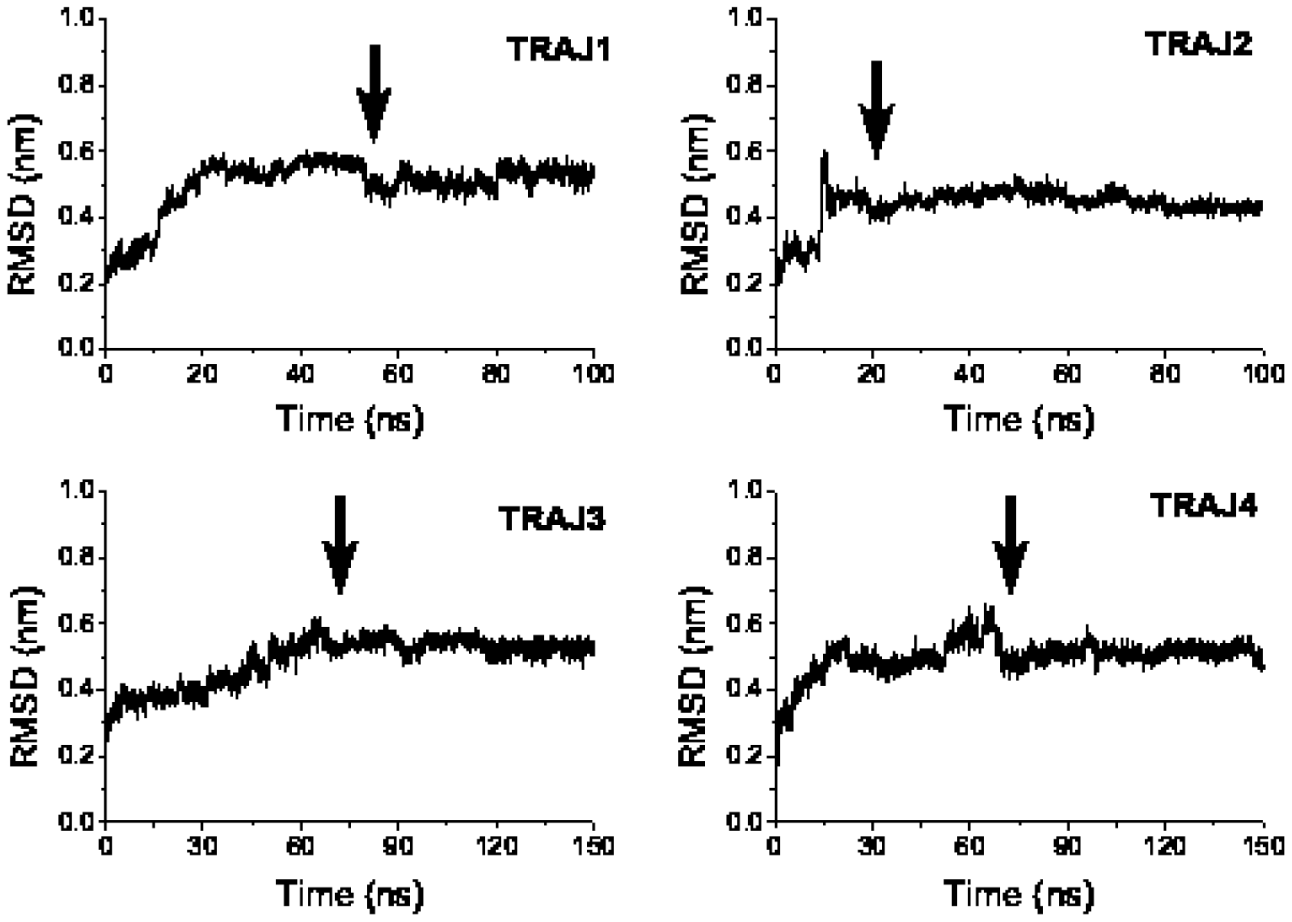
Time dependence of root mean square deviation of insulin in complex with RGFFYT. The time dependence of C*_α_*-rmsd of insulin in complex with RGFFYT from its initial conformation obtained in four MD simulations. Arrows roughly refer to equilibration time 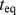 when the curve starts to saturate. 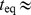
55, 20, 70, and 70 ns for trajectories 1, 2, 3, and 4, respectively.

In order to have more reliable estimation of binding energy we apply the MM-PBSA method to insulin-RGFFYT complex. MD simulations with the Gromacs force field 43a1 and SPC water model have been performed using the best docking conformation (Fig. 8) as the initial conformation. Four 100–150 ns trajectories were generated using different random seed numbers and time dependence of C*_α_*-rmsd of insulin from its initial conformation is shown in Fig. 9. As in the insulin-LVEALYL case, the system reaches equilibrium at different time scales for different MD runs.

The receptor-ligand interaction energies which include the electrostatic and vdW interactions are sensitive to MD runs (Figure S15). As a result, the binding free energy fluctuates among 4 trajectories (Table 1). However, apolar solvation energy 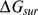 and entropy contribution are not sensitive to them remaining lower than those for the LVEALYL cases. This is probably RGFFYT has one residue less than LVEALYL.

As in the case of LVEALYL the electrostatic interaction is more important than vdW interaction but the difference between these two interactions is much more pronounced. Within error bars the vdW contributions are the same for insulin-RGFFYT and insulin-LVEALYL complexes (Table 1). However, the Coulomb interaction between RGFFYT and insulin is much stronger. This is associated with the fact that negatively charged insulin has more attractive interaction with positively charged RGFFYT (residue E) than with LVEALYL having negatively charged residue E.

Within error bars RGFFYT and LVEALYL have the same 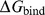 (Table 1) and the inhibition constant IC_50_∼nM. Therefore, similar to LVEALYL, RGFFYT may be used to interfere aggregation process as it shows high binding affinity towards insulin.

### Self-assembly of RGFFYT

**Figure 10:**
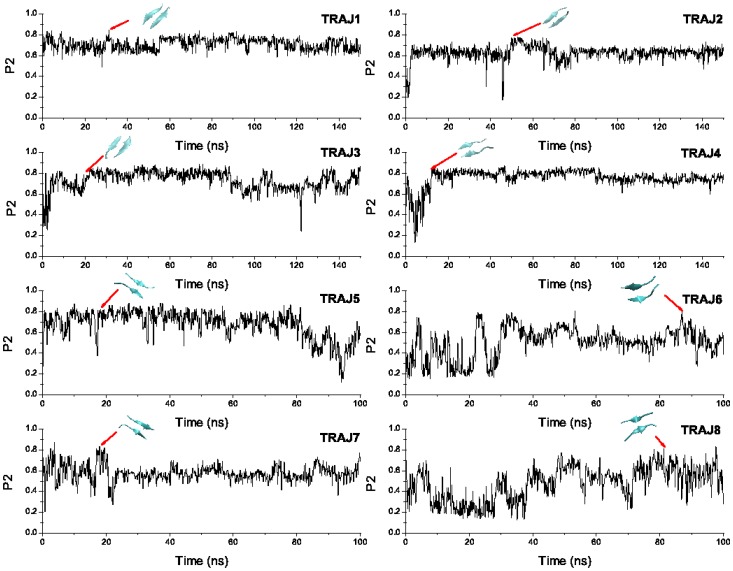
Time dependence of order parameter 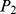 for dimer of RGFFYT. The same as in Figure 1 but for RGFFYT. Snapshots show fibril conformations that occur for the first time. The first passage time is 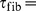
33.1, 51.8, 20.0, 12.0, 19.0, 87.0, 19.3, and 81.2 ns for trajectory 1, 2, 3 4, 5, 6, 7, and 8, respectively.

To check if RGFFYT can aggregate we have performed 8 MD simulations at 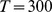 K for dimer starting from random configurations. As follows from the time dependence of the order parameter 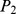, the anti-parallel fibril conformation occurs for the first time at 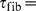
33.1, 51.8, 20.0, 12.0, 19.0, 87.0, 19.3, and 81.2 ns for trajectory 1, 2, 3 4, 5, 6, 7, and 8, respectively (Fig. 10). Because this peptide has one charged residue R at the N-terminal, the antiparallel ordering of dimer is favorable to minimize the inter-chain electrostatic interaction. One can show that in the fibril state two chains form the beta sheet having the number of backbone-backbone HBs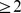 (Figure S2). Having the average fibril formation time 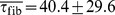 ns one can expect that RGFFYT forms dimer a bit faster than LVEALYL. This is not surprising because RGFFYT has one residue less and shorter peptides are supposed to form fibril faster. Another possible reason for rapid oligomerization of RGFFYT is that the charged residue R is at the end of peptide, while negatively charged residue E of LVEALYL is located in the middle.

To get more insight on capability of two peptides to fibrillation we use Zyggregator method [57], [58] for prediction of intrinsic aggregation propensities (Zagg). The result obtained for insulin is shown in Figure S16. Averaging over residues we obtain 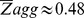, and 0.86 for LVEALYL residues B11-17 and RGFFYT residues B22-27, respectively. Therefore RGFFYT shows higher propensity to aggregation than LVEALYL and this is in line with our MD simulation on oligomerization rates. Note that fragments from region A4-A13 (Figure S16) may be highly fibril-prone having high Zagg values but this issue has not been addressed. It would be interesting to study the binding affinity to insulin and self-assembly properties of short peptides from this region.

## Conclusions

Through all-atom simulations have obtained the following results.

1. We have found that insulin monomer is very stable and this observation is consistent with Pease III's experimental result [48] that insulin wouldn't unfold itself until it attaches with the end of amyloid fibril according to their study in insulin oligomer.
2. It is shown that LVEALYL does not bind to itself in insulin but to fragment B22-27 or to peptide RGFFYT. The binding is rather driven by the electrostatic interaction than the vdW interaction because both ligand and receptor are charged.
3. At pH = 7 dimer LVEALYL can form fibril structure with antiparallel ordering. This is probably valid for much larger beta sheets because such a ordering is favored by charged residue Glutamic acid at the third position. Using MD simulation and the Zyggregator method for calculation of propensity profile [57], [58] we predict that RGFFYT can also self-ensemble and its propensity to aggregation may be even higher than LVEALYL. Antiparallel ordering of fibril state of both peptides is presumably associated with the existence of charged residues in their sequences.
4. Using the MM-PBSA method one can show that that the inhibition constant IC50 of LVEALYL and RGFFYT is of nM. The high binding affinity of RGFFYT would allows it to modulate fibril formation of insulin. It would be interesting to check this prediction by experiments.
5. Within present computational facilities, it is difficult to probe the effect of LVEALYL on fibril growth of insulin at atomic level. However, our study indicates that this peptide affects aggregation process through the binding mechanism.

## Supporting Information

Information S1 **MM-PBSA method.** The MM-PBSA method used to calculate the binding free energy of ligand to receptor is described in detail.
(PDF)

Figure S1
**One-dimensional FEL for LVEALYL dimer.** The free energy is plotted as a function of 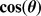. Clearly, the probability of observing parallel ordering with 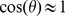 is much lower than antiparallel arrangement. Results were obtained from 8 MD trajectories shown in Fig. 1 of the main text.(EPS)

Figure S2
**Inter-chain backbone-backbone HBs for LVEALYL and RGFFYT dimers.** (A) Seven inter-chain backbone HBs of dimer LVEALYL in the fibril state with order parameter 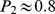. The snapshot was taken from trajectory 4 in Fig. 1 of the main text. (B) The same as (A) but for RGFFYT dimer (trajectory 6 from Fig. 10 of the main text), where one has 2 backbone HBs. Red, white, cyan and blue balls are O, H, N and C atoms.(EPS)

Figure S3 **Contact maps for the interaction energies of LVEALYL dimer.** The interaction energies (vdW and electrostatic terms) between residues from two chains of LVEALYL dimer. The results were obtained using data obtained from 8 MD trajectories. The color bar on the right hand side refers to the interaction energy that is measured in kcal/mol.
(EPS)

Figure S4 **Contact maps for LVEALYL dimer.** Hydrogen bond (A) and side chain (B) contact map for dimer LVEALYL. The results were obtained using data obtained from 8 MD trajectories. The color bar on the right hand side refers to the probability of formation of inter-peptide contacts.
(EPS)

Figure S5 **Insulin structures at pH 2.1 and 7.** The PDB structure 1GUJ obtained at pH = 2.1 (blue), while red color refers to the most populated cluster structure. The rmsd between two structures is 0.28 nm.
(EPS)

Figure S6 **Insulin structures at pH 8.5 and 7.** The PDB structure 3I3Z obtained at pH = 8.5 (blue), while red color refers to the typical structure of the most populated cluster. The rmsd between two structures is 0.26 nm.
(EPS)

Figure S7 **Secondary structure of insulin from MD trajectory 1.** Time dependence of second structure contents of insulin in the absence (black) and presence (red) of LVEALYL (fragment B11-17). The results have been obtained using STRIDE definitions for secondary structures and snapshots collected in MD trajectory 1.
(EPS)

Figure S8 **Secondary structure of insulin from MD trajectory 2.** The same as in Fig. S7 but for the second MD trajectory.
(EPS)

Figure S9 **Secondary structure of insulin from MD trajectory 3.** The same as in Fig. S7 but for the third trajectory.
(EPS)

Figure S10 **Secondary structure of insulin from MD trajectory 4.** The same as in Fig. S7 but for the fourth MD trajectory.
(EPS)

Figure S11

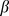
 **-content of insulin in complex with LVEALYL.** The results have been obtained for two time windows 50–150 ns and 50–300 ns using snapshots collected in four MD runs.(EPS)

Figure S12

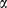
 **-helix contents of B11-17 fragment.** The helix content of this fragment within insulin in the absence (black) and presence (red) of LVEALYL. The results were obtained in equilibrium of four 300 ns MD simulations.(EPS)

Figure S13 **Time dependence of the length of beta structure of fragment B22-27.** The results are shown for the first trajectory of 300 ns MD simulation.
(EPS)

Figure S14 **Time dependence of HBs between insulin and LVEALYL.** Black refers to the total number of HBs, while red denotes the number of HBs that are available in the best docking conformation. The result was obtained for trajectory 1.
(EPS)

Figure S15 **Time dependence of interaction energy between insulin and RGFFYT.** The results are shown for 4 MD trajectories. Arrows roughly refer to time of reaching equilibrium (see Fig. 9 in the main text).
(EPS)

Figure S16
**Aggregation propensity profile of insulin.** Vertical bars refer to the intrinsic aggregation propensity 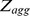. Fragments B11-17 (LVEALYL) and B22-27 (RGFFYT) are colored in green and red, respectively.(EPS)

Movie S1 **Dynamics of LVEALYL during MD simulation.** This movie shows the movement of peptide LVEALYL around insulin during 300 ns MD run.
(ZIP)
