# Supplementary material for: Oligomerization of Peptides LVEALYL and RGFFYT and Their Binding Affinity to Insulin
Source: PLoS One. 2013 Jun 21;8(6):e65358. doi: 10.1371/journal.pone.0065358 (PMC3689759; doi:10.1371/journal.pone.0065358)
Supplement: Information S1 — MM-PBSA method. The MM-PBSA method used to calculate the binding free energy of ligand to receptor is described in detail. (PDF) [file pone.0065358.s001.pdf]

MM-PBSA method. The binding free energy is defined by the following equation

$$\Delta G_{\text{bind}} = G_{\text{complex}} - G_{\text{free-protein}} - G_{\text{free-ligand}}. \tag{S1}$$

The MM-PBSA approach is summarized by the following equations

$$\begin{aligned} G &= E_{\text{gas}} + G_{\text{solvation}} - TS, \\ G_{\text{solvation}} &= G_{\text{PB}} + G_{\text{sur}}, \\ G_{\text{sur}} &= \gamma A + b. \end{aligned} \tag{S2}$$

The energy in the gas phase E_{gas} is as follows

$$E_{\text{gas}} = E_{\text{bond}} + E_{\text{angle}} + E_{\text{tors}} + E_{\text{vdw}} + E_{\text{elec}}. \tag{S3}$$

Local terms E_{bond}, E_{angle} and E_{tors} come from covalent bonds, bending, and torsion interactions. E_{vdw} and E_{elec} are the van der Waals and the electrostatic interaction energies, respectively. Total molecular mechanical energies E_{gas} are calculated by using GROMACS utility with the same force field used in the MD simulations, but no cut-off is used for the evaluation of nonbonded interactions.

$G_{\text{solvation}}$ represents the free energy of solvation and TS is the solute entropic contribution. $G_{\text{solvation}}$ consists of two parts, the polar solvation (G_{PB}) and nonpolar solvation energy (G_{sur}). G_{PB} arises from the electrostatic potential between solute and solvents and it is determined by using the continuum solvent approximation [1]. The APBS software package [2] was employed for numerical solution of the corresponding linear Poisson-Boltzmann equation. The cubic lattice has a grid spacing of 0.5 Å. The GROMOS radii and charges were used to generate the PQR files. The continuum medium is assumed to have the dielectric constant of water with no salt $\epsilon = 78.54$ and the solute dielectric constant $\epsilon = 2$. The surface or nonpolar solvation term G_{sur} is defined by the solvent-accessible surface area A and two empirical parameters $\gamma = 0.0072$ kcal/(mol.Å^2) and $b = 0$ [3]. Here A was estimated using the Shrake-Rupley numerical approximation [4] implemented in the APBS package.

In the MM-PBSA approximation, snapshots collected from the MD run for the protein-ligand complex are used for estimating $G_{\text{free-protein}}$ and $G_{\text{free-ligand}}$. After equilibration, snapshots of complex, protein and ligand (without water) were taken every 10 ps for calculating the enthalpy.

Solute entropy contributions were estimated for the snapshots that were taken every 100 ps from equilibration MD runs. The structures were minimized with no cutoff for nonbonded interactions by using conjugate gradient and low-memory Broyden–Fletcher–Goldfarb–Shanno method [5] until the maximum force was smaller than 10^{-6} kJ/(mol.nm). Normal mode analysis was performed by calculating and diagonalizing the mass-weighted Hessian matrix. The frequency of the normal mode was then used to calculate the vibration entropy [6] as given by the following equation

$$S_{\text{vib}} = -R\ln(1 - e^{-h\nu_\circ/k_BT}) + \frac{N_A \nu_\circ e^{-h\nu_\circ/k_BT}}{T(1 - e^{-h\nu_\circ/k_BT})}, \tag{S4}$$

where S_{vib} is the vibrational entropy, h Plank's constant, $\nu_\circ$ the frequency of the normal mode, k_B the Boltzmann constant, $T = 300$ K, and N_A Avogadro's number. The total vibration entropy is a sum over all frequencies of the normal mode analysis.

References

1. Sharp KA, Honig B (1990) Electrostatic interactions in macromolecules: theory and applications. Annu. Rev. Biophys. Biophys Chem 19: 301–332.

2. Baker NA, Sept D, Joseph S, Holst MJ, McCammon JA (2001) Electrostatics of nanosystems: application to microtubules and the ribosome. Proc Natl Acad Sci USA 98: 10037–10041.

3. Sitkoff D, Sharp KA, Honig B (1994) Accurate calculation of hydration free energies using macroscopic solvent models. J Phys Chem 97: 1978-1988.

4. Shrake A, Rupley JA (1973) Environment and exposure to solvent of protein atoms-lysozyme and insulin. J Mol Biol 79: 351-371.

5. Shanno DF (1970) Conditioning of quasi-newton methods for function minimization. Matthematics of Computation 24: 647-656.

6. McQuarrie DA (1973) Statistical Thermodynamics. New York: Harper and Row, 2 edition.
